# Neural representation of a spatial odor memory in the honeybee mushroom body

**DOI:** 10.1186/1471-2202-16-S1-P240

**Published:** 2015-12-18

**Authors:** Martin P Nawrot, Tiziano D'Albis, Randolf Menzel, Martin Strube-Bloss

**Affiliations:** 1 Bernstein Center for Computational Neuroscience Berlin, Berlin, Germany; 2 Computational Systems Neuroscience, Department of Biology, University of Cologne, Cologne, Germany; 3 Institute of Biology - Neurobiology, Freie Universität Berlin, Berlin, Germany; 4 Department of Behavioral Physiology & Sociobiology, Biocenter, University of Würzburg, Würzburg, Germany

## 

Insects make use of lateralized olfactory information from their left and right antennae. Honeybees can learn to distinguish side-specific odor cues in classical conditioning experiments, i.e. they associate a specific stimulus combination of odor identity and spatial location (left or right) with the reward [1]. This requires inter-hemispheric transfer of lateralized information and a side-specific odor memory. Mushroom body (MB) output neurons, the so-called extrinsic neurons (ENs), make inter-hemispheric connections between the two MBs and are thus candidates for the inter-hemispheric transfer of lateralized stimulus information. We could show previously that ENs in the honeybee undergo plastic changes in classical conditioning [2].

Here, we investigate neuronal plasticity in ENs of the honeybee in a sides-specific learning paradigm. We performed multiple single-unit recordings from ENs of one MB. Prior to conditioning (PRE) each bee was repeatedly presented with two different odors on the two antennae separately. During acquisition one of these odors was repeatedly presented to the antenna contralateral to the recording side (CS+) and paired with a sugar reward, while the other odor was presented without reward (differential conditioning). Three hours after training (POST) we repeated the initial protocol presenting each of the two odors repeatedly to each of the antennae. In the behavioral test the bees distinguished the CS+ stimulus configuration of odor and side from the other three stimulus combinations.

At the neuronal level we found clear and distinct odor representations in the EN population before training (PRE) only when stimulated on the antenna *ipsilateral *to the recording side. However, no odor responses were measured in any of the ENs when stimulated at the *contralateral *antenna. This picture changed drastically after training (POST). Now, the rewarded stimulus combination (CS+) resulted in a strong population response pattern. The population code for the CS+ configuration was clearly distinct from all three other stimulus configurations. Quantification of the temporal response latencies showed that the ENs encode an odor approximately within 75ms for ipsilateral stimulation. Odor representation was delayed by about 60ms for a contralateral stimulation. We discuss two alternative explanations for this temporal delay.

We hypothesized previously that ENs at the MB output encode the experience-dependent *value *of a particular stimulus [2-4]. Our results here provide additional evidence for this hypothesis. A representation of the rewarded stimulus combination (CS+) of a particular odor and its spatial location (left or right) develops only due to reward conditioning. Before learning, only ipsilateral odor information was represented.
